# Ticks and tick-borne diseases in the northern hemisphere affecting humans

**DOI:** 10.3389/fmicb.2025.1632832

**Published:** 2025-08-08

**Authors:** Nathalie Boulanger, Hayato Iijima, Kandai Doi, Yuya Watari, Mackenzie Kwak, Ryo Nakao, Stephen Wikel

**Affiliations:** ^1^UR 3073: PHAVI: Groupe Borrelia, Institut de Bactériologie, University of Strasbourg, Strasbourg, France; ^2^French National Reference Center for Borrelia, Hôpitaux Universitaires, Strasbourg, France; ^3^Department of Wildlife Biology, Forestry and Forest Products Research Institute, Tsukuba, Japan; ^4^Laboratory of Parasitology, Graduate School of Infectious Diseases, Faculty of Veterinary Medicine, Hokkaido University, Sapporo, Japan; ^5^Division of Parasitology, Veterinary Research Unit, International Institute for Zoonosis Control, Hokkaido University, Sapporo, Japan; ^6^One Health Research Center, Hokkaido University, Sapporo, Japan; ^7^Department of Medical Sciences, Frank H. Netter, M. D., School of Medicine, Quinnipiac University, Hamden, CT, United States

**Keywords:** ticks, tick-borne diseases, climate change, invasive ticks, socio-ecosystems, prevention, management, One Health

## Abstract

Temperate zones of the northern hemisphere are increasingly impacted by human biting ticks and the human pathogens they transmit. The relationships among ticks, hosts, and pathogens are undergoing significant changes with consequences for human health. This northern hemisphere focused review examines human biting ticks and the disease causing agents they transmit as increasing public health threats due to geographic range expansion, increasing size of tick populations, emergence of newly recognized pathogens, introduction of invasive tick species that are resulting in part from changing weather patterns, land use modifications, biodiversity loss, and human activities/behaviors; all of which result in significant challenges for tick control and disease prevention. As a result of these evolving interactions and the resulting threats they pose, there exist critical needs to implement existing and develop novel tools and strategies to prevent tick bites, control tick populations, and reduce transmission of tick-borne pathogens. Timely, up to date knowledge of which ticks and tick-borne infectious agents are present within an area is foundational for physicians, public health authorities tasked with disease prevention, and the public. Achieving these objectives poses significant challenges. Here, we examine current medically important tick – host - pathogen relationships in Asia, Europe, and North America.

## Ticks and tick-borne diseases situation

Emerging, re-emerging, and endemic infectious diseases are increasing threats in this era of global change in part due to climate change (Baker et al., 2022; Semenza et al., 2022). Vector-borne diseases are particularly susceptible to climate variations (Campbell-Lendrum et al., 2015; Thomson and Stanberry, 2022). Amongst disease vector arthropods, ticks transmit the greatest diversity of potentially pathogenic microorganisms such as bacteria, protozoa, and viruses (Colwell et al., 2011; Estrada-Peña and Jongejan, 1999; Jongejan and Uilenberg, 2004). Human biting ticks and the disease causing agents they transmit are increasingly challenging global public health threats due to their expanding geographic ranges; appearance and establishment of invasive tick species; emergence and resurgence of potential pathogens; changing vertebrate host populations; limited effective tick control tools and strategies; absence of anti-tick and anti-pathogen vaccines; and, changing biotic and abiotic factors that influence tick-host-pathogen relationships (Beard et al., 2019; Boulanger et al., 2024; Dantas-Torres, 2015; Eisen and Stafford, 2021; Sonenshine, 2018; Tsao et al., 2021; van Oosterwijk and Wikel, 2021; Wikel, 2022).

Climate impacts the seasonal dynamics of ticks. The ixodid tick life cycle involves free-living periods of months to years interspersed with brief periods for days of host attachment and blood feeding (Sonenshine, 2018). Free-living life cycle stages are susceptible to climate variations that influence tick population sizes, geographic ranges, pathogens transmitted, and their interactions with other tick species that are unique from those of other disease vector arthropods (Estrada-Peña, 2015; Ogden and Lindsay, 2016). Additional factors that influence tick communities are vegetation, land use, habitat modification, and changes in animal host communities. Environmental warming occurring in the northern hemisphere will drive tick geographic range expansions further northward and to higher altitudes, while concurrent range contractions could occur in subtropical and tropical regions. Data regarding geographic range changes are becoming available for some of the more significant human biting ixodid tick vectors in Asia, Europe, and North America. Range expansions of human biting ixodid ticks into new territories are introducing previously unencountered pathogens and creating potential changes in tick ecologies, balance among tick species, and tick-host associations that present new challenges for tick control, disease prevention, and interventions (Eisen, 2020a; Sonenshine, 2018; Wikel, 2022). Tick-transmitted infections are zoonotic diseases (Pfäffle et al., 2013). This diverse community of infectious agents is not static as evidenced by the increasing frequency of emergence of newly discovered zoonotic pathogens during this time of unprecedented global geoclimatic, demographic, socioeconomic, and technological changes (Rupasinghe et al., 2022; Skowron et al., 2023).

Knowing where ticks and tick-borne pathogens are present is foundational to understanding the health threats they represent. Ranges of human biting ticks and tick-borne pathogens are changing and thus creating further gaps in our already fragmented knowledge of where these vectors and pathogens are present (Eisen and Paddock, 2021). Surveillance of tick populations is needed on an ongoing basis combined with next generation sequencing of tick microbiomes (bacteria, viruses, eukaryotic parasites and fungi, commensal and pathogenic) to identify and link to specific geographic areas established, resurging, emerging, and potential human pathogens (Osikowicz et al., 2024). Concomitant with these surveillance strategies must be development of broadly searchable, accessible databases on tick and tick-borne pathogens. Discussions are occurring about development of national strategies relating to surveillance, control, knowledge gaps, interventions, and development of new management tools (Beard et al., 2019; Eisen and Stafford, 2021).

This review draws together in one document essential information on the current distributions of ticks, tick-borne pathogens, and pathogen reservoir species in the northern hemisphere that will further identify knowledge gaps regarding tick and pathogen biology and ecology, intervention measures and control strategies, and need for government support for such endeavors to support public health and the wellbeing of society at large.

## Main tick species on each continent

Ticks are arachnids of the subclass Acari within the monotypic order Ixodida. There are three families of ticks, though only two are medically important: The Ixodidae (hard ticks) and the Argasidae (soft ticks) (Supplementary Table 1). The Nuttalliellidae is monotypic, only present in Africa, and not observed for decades; while the Argasidae are species rich with approximately 200 species, and distributed on all continents, except Antarctica (Guglielmone et al., 2010). The hard ticks have a worldwide distribution with 683 species, of which 283 species bite humans (69 Prostriata and 214 Metastriata) (Guglielmone and Robbins, 2018). The hard ticks are divided into two groups: Prostriata containing the genus *Ixodes* and Metastriata with 14 genera (Guglielmone and Robbins, 2018).

Ticks are strictly hematophagous, feeding on various vertebrate hosts: amphibians, reptiles, birds, and mammals. Hard ticks are characterized by long feeding periods of days, duration dependent upon life cycle stage (larva, nymph, adult), whereas soft ticks typically feed for short durations of minutes to hours and repeatedly (de La Fuente et al., 2008). Hard ticks have 4 life stages (eggs, larvae, nymphs and adults), of which 3 are active. Their complete development can occur within 3 weeks for *Rhipicephalus microplus* (Oyen and Poh, 2025), or more typically a year, or several years depending on the species and local abiotic (e.g., temperature and humidity) and biotic (e.g., host availability) factors. Species can be either nidicolous (living in nests, burrow or wall cracks), or non-nidicolous (on vegetation or leaf litter in the environment), which is more conducive to exposure to humans.

In the United States, 36 ixodid species and 13 argasid species have been recorded infesting humans (Eisen, 2022). The four main medically important hard tick genera are *Ixodes*, *Amblyomma, Dermacentor*, and *Rhipicephalus* with the invasive species *Haemaphysalis longicornis* an increasing threat as a human biter. In Europe, around 40 tick species potentially biting humans have been identified with *Ixodes* and *Dermacentor* being the most medically important (Estrada-Pena et al., 2017; Perez et al., 2023; Rubel et al., 2018). Asia has a significantly more species-rich tick community than both Europe and the United States combined with more than 100 species of which most belong to the genera *Dermacentor*, *Ixodes*, and *Haemaphysalis* (Zhao et al., 2021).

***Ixodes*, the largest tick genus**, includes more than 250 species (Estrada-Pena et al., 2017; Guglielmone and Robbins, 2018; Keirans and Durden, 2014). The *Ixodes ricinus* complex consists of at least 15 species with some of the most notable being *I. ricinus* and *I. persulcatus* in Eurasia, and *I. scapularis*, and *I. pacificus* in North America. Human biting species in Asia include *I. granulatus*, *I. nipponenis*, *I. pavlovskyi* and *I. sinensis*.

*Ixodes* are three host ticks which often exhibit non-nidicolous behavior as adults. However, the immature stages of some *Ixodes*, such as *I. ovatus*, are nidicolous. Many medically important *Ixodes* species are non-nidicolous generalists. Their biology and ecology are strongly influenced by temperature, humidity, habitat change, and host availability, all of which influence tick range expansion and abundance (Cunze et al., 2022; Medlock et al., 2013). Nymphs are the most common stage infesting humans (Barbour and Fish, 1993). The *I. ricinus* complex is abundant in deciduous and coniferous forested areas with relatively high humidity and increasingly in urban and suburban areas (Diuk-Wasser et al., 2010; Kahl and Gray, 2023; Rizzoli et al., 2014; VanAcker et al., 2019).

The geographic ranges of *I. ricinus* and *I. persulcatus* are expanding with *I. ricinus* in Europe and *I. persulcatus* in Asia with overlap in some interfacing regions (Bugmyrin et al., 2022). The range of *I. ricinus* extends from the Ural Mountains to Ireland and from northern Africa to Scandinavia (Estrada-Peña et al., 2012). It has expanded in northern Sweden and become more abundant in central and southern Sweden during the past three decades (Jaenson et al., 2012a). Range extension of *I. ricinus* northward in Norway is attributed to increased mean temperatures over the past decade (Hvidsten et al., 2020). As a result, the vegetation growing season is longer. Over 170 days, this approximate index is associated with sites of permanent tick populations (Hvidsten et al., 2020). In addition to range extensions at the previously recognized limits of altitude and latitude, *I. ricinus* distribution is changing within its established range (Medlock et al., 2013).

Range expansions are also occurring for multiple medically important North American tick species (Wikel, 2022; Table 1). *Ixodes scapularis*, the most important vector of Lyme borreliosis in North America, doubled the number of counties in the United States in which it was established in the 20 years period from 1996 to 2016 (Eisen et al., 2016). Northward expansions of the geographic range of *I. scapularis* in the eastern and midwestern United States resulted in established populations in Ontario and Quebec, Canada (Robinson et al., 2022; Scott and Pesapane, 2021; Slatculescu et al., 2020). *Ixodes scapularis* is reclaiming its historic range across a broad area of eastern regions of midwestern North America (Eisen and Eisen, 2018).

**TABLE 1:** Main tick-borne diseases and associated pathogens affecting human according to the continent (Zhao et al., 2021).

Diseases/vectors	Asia: China/Japan	Europe	United States/Canada
**Bacterial infections**			
Lyme disease *Ixodes ricinus* complex	*B. afzelii* *B. garinii* *B. burgdorferi*s.s. *B. valaisiana-like* *B. bavariensis*	*B. afzelii* *B. garinii* *B. burgdorferi* ss	*B. burgdorferi*s.s. *Borrelia mayonii*
Anaplasmosis *Ixodes ricinus* complex	*Anaplasma phagocytophilum*	*Anaplasma phagocytophilum*	*Anaplasma phagocytophilum*
Hard tick relapsing fever *Ixodes ricinus* complex****	*B. miyamotoi*	*B. miyamotoi*	*B. miyamotoi*
Neoehrlichiose *Ixodes ricinus* complex	*N. mikurensis*	*N. mikurensis*	(−)
Rickettsioses *Ixodes ricinus* complex *Dermacentor* spp. *Rhipicephalus sanguineus*complex *Amblyomma* spp. *Haemaphysalis* spp.	*R. heilongjiangensis* *R. raoultii* *R. japonica* *R. monacensis* *R. helvetica*	*R. conorii* *R. raoultii* *R. slovaca*	*R. rickettsii*
Soft tick relapsing fever *Ornithodoros* spp.	−	(−)	*Borrelia hermsii* *B. parkeri* *B. turicatae*
Ehrlichioses *Amblyomma*spp.	*E. chaffeensis* *E. ewingii* *E. muris eauclairensis*	(−)	*E. muris eauclairensis* *E. chaffeensis* *E. ewingii*
Tularemia *Ixodes ricinus* complex *Dermacentor* spp. *Amblyomma* spp.	F. tularensis*	F. tularensis*	F. tularensis*
Q fever *Ixodes ricinus* complex *Hyalomma* spp. *Dermacentor* spp. *Haemaphysalis* spp.	C. burnetii*	C. burnetii*	C. burnetii*
**Viral infections**			
Powassan virus infection *Ixodes ricinus* complex	−	−	+
Tick-borne encephalitis *Ixodes ricinus* complex****	+	+	−
Congo Crimean hemorrhagic fever *Hyalomma spp.*	+	+	−
Bourbon virus infection *Amblyomma*spp.	−	−	+
Heartland virus infection *Amblyomma*spp.	−	−	+
Severe fever with thrombocytopenia syndrome virus (SFTSV) infection *Haemaphysalis* spp.	+	(−)	(−)
Jingmen virus infection *Ixodes ricinus* complex *Rhipicephalus*spp.	+	+	−
Alongshan virus infection *Ixodes ricinus* complex	+	+	−
**Parasitic infections**			
Babesioses *Ixodes ricinus* complex	*B. microti* *B. divergens* *B. venatorum* *B. crassa-like*	*B. divergens* *B. venatorum*	*B. microti* *B. duncani*

*Some pathogens, particularly, *Francisella* and *Coxiella*, have other main modes of transmission. +: presence, (−): absence.

As the primary vector of Lyme borreliosis and tick-borne encephalitis in Eurasia (Table 1), the genus *Ixodes* receives the most attention. In addition, more recently, different pathogens have been associated with this genus such as *Babesia* spp.*, Anaplasma phagocytophilum*, *Neoehrlichia mikurensis*, and *Borrelia miyamotoi*. *Ixodes* ticks and their associated pathogens’ distribution is likely shifting across much of the world due to climate change, ecosystem modifications, and socio-economic changes (Eisen, 2022; Kahl and Gray, 2023; Sprong et al., 2018).

**The genus *Dermacentor*** includes around 40 species in the Palearctic, Oriental, Afrotropical, and Nearctic zoogeographic regions with five species particularly relevant to human medicine in the Northern hemisphere: *Dermacentor variabilis* (American dog tick), *Dermacentor andersoni* (Rocky Mountain wood tick), *Dermacentor occidentalis* (Pacific Coast tick), *Dermacentor marginatus* (Eurasia) and *Dermacentor reticulatus* (Eurasia) (Guglielmone and Robbins, 2018). Most are three-host ticks, though some species engage in facultative two-host life cycles. *Dermacentor* species are predominantly mammalian ectoparasites, although immature stages are sometimes also recorded on birds. The immature stages are often endophilic, feeding on small mammals, adults predominantly obtain blood meals from large ruminants. Major medically important species are *D. reticulatus* and *D. marginatus* in Eurasia (Földvári et al., 2016; Garcia-Vozmediano et al., 2020), *D. andersoni* and *D. variabilis* in the United States and Canada (Eisen, 2022), and *D. belullus* and *D. auratus* in Asia (Apanaskevich and Apanaskevich, 2015; Kwak et al., 2021; Supplementary Table 1). They are important vectors of rickettsial infections worldwide. *Dermacentor andersoni* and *D. variabilis* are vectors of Rocky Mountain spotted fever (RMSF) in United States (Keirans and Durden, 2014), while *D. reticulatus* and *D. marginatus* are potential vectors of tick-borne lymphadenopathy in Europe (Chitimia-Dobler et al., 2019; Keirans and Durden, 2014). Tick-borne lymphadenopathy is caused by spotted fever group rickettsia, frequently *Rickettsia slovaca* (Silva-Pinto et al., 2014). In Asia, *D. auratus* has been implicated in the transmission of Kyasanur forest disease (Kwak et al., 2021). *D. auratus* is frequently encountered in both forests and urban parklands due to its close association with wild pigs (*Sus scrofa*), which act as the major reservoir host for the Kyasanur virus (Teo et al., 2024). *D. reticulatus* is an important vector of human pathogens that is significantly expanding its range in Europe (Cunze et al., 2022; Földvári et al., 2016). While climate change is a major driver, this highly adaptable species continues to establish populations in multiple new regions of Europe, likely in response to changing land use, and the widespread abandonment of previously approved tick control chemicals (Dautel et al., 2006; Piesman and Eisen, 2008; Randolph, 2010). Ecological modeling predicts range expansion of both *D. reticulatus* and *D. marginatus* toward northeastern Europe, more widely in central Europe, and the existence of areas where these species are sympatric (Cunze et al., 2022). In North America, the northern distribution limit in the prairie of southwestern Canada remains stable for *D. andersoni*, a significant medically important western North American tick, while expansion to the north and west by the closely related *D. variabilis*, an eastern North American species, results in overlapping ranges of these two species within an approximately 200 kilometer zone (Dergousoff et al., 2013).

**The genus *Rhipicephalus*** contains around 75 species; however, there exists uncertainty regarding the identity of certain species (Dantas-Torres and Otranto, 2015). We will focus on the *R. sanguineus* complex, which is the most widely distributed tick taxon. It is mainly associated with domestic dogs. Members of the complex are generally regarded to be three host ticks. Their development can be rapid, sometimes far less than a year (2–4 months) under warm climates and this species is commonly associated with dog shelters and human habitats where these ticks hide in wall crevices (Dantas-Torres, 2010). All stages can bite humans; and, they are reported to be more aggressive under warmer temperatures. *Rhipicephalus sanguineus* s.l. is considered to be an important vector of the rickettsial infections, Mediterranean Spotted fever (MSF) in Europe and Rocky Mountain spotted fever in Mexico and the southwestern United States (Dantas-Torres and Otranto, 2015).

**The genus *Amblyomma*** contains around 138 species mainly in the Neotropical, Afrotropical, Oriental, and Australasian zoogeographic regions (Guglielmone and Robbins, 2018). However, in North America, *A. americanum*, larvae, nymphs, and adults of the lone star tick, frequently bite humans (Eisen, 2022). It is a generalist species with all active stages (larva, nymph, and adult) feeding on all classes of terrestrial vertebrates. It is the vector of tularemia, RMSF, Q fever, and ehrlichiosis (*E. chaffeensis*) (Keirans and Durden, 2014). *A. americanum* is also known as the vector of STARI, Southern Tick Associated Rash Illness, *Ehrlichia ewingii*, Heartland virus and bourbon virus. Both viruses are emerging infectious diseases in North America (Dupuis et al., 2023). *A. americanum* is an aggressive human-biting tick that is expanding from its traditional range in the southeastern United States into the mid-Atlantic, northeastern, and midwestern United States and into southern Canada (Gasmi et al., 2018; Molaei et al., 2019; Sagurova et al., 2019; Sonenshine, 2018; Springer et al., 2015). *A. maculatum*, vector of the spotted fever rickettsia, *R. parkeri*, is also expanding from its historic range along the coastal southeastern United States northward into the mid-Atlantic, southern New England, and significantly further inland along its range (Bajwa et al., 2022; Paddock and Goddard, 2015; Teel et al., 2010). Isolated populations of *A. maculatum* occur at multiple locations in the midwestern and southwestern United States, well beyond the traditional range for this tick species (Paddock and Goddard, 2015). *A. testudinarium*, *A. kappa*, and *A. helvolum* are also known to bite humans in Asia. *A. testudinarium* in particular is a vector of the zoonotic Oz virus and *R. tamurae*. It is also suspected to be a vector of Severe Fever with Thrombocytopenia Syndrome (SFTS) virus and *Hepatozoon* spp. (Nakao et al., 2021). *A. testudinarium* is the main species responsible for human tick-bite cases in Japan, and the main *Amblyomma* species biting humans throughout East and Southeast Asia (Natsuaki, 2021). The study of tick bites in wild animals and humans showed that the expansion of wild boar was to blame, and that the majority of these cases were associated with *A. testudinarium* (Shimada et al., 2022). This tick is also linked to cases of alpha-gal syndrome, a hypersensitivity reaction to red meat mediated by IgE antibody (Hashizume et al., 2018).

**The genus *Haemaphysalis*** includes around 166 species with a cosmopolitan distribution (Guglielmone and Robbins, 2018). Its highest species diversity occurs in East and Southeast Asia, less so in Europe and the Americas (Durden and Beati, 2014; Guglielmone and Robbins, 2018). *Haemaphysalis longicornis* in China, Japan, and Korea is a vector of SFTS virus, a significant emerging infectious disease. Presently, this tick species is considered an invasive tick in many regions. Originally native to East Asia, it spread to Australia, New-Zeland and many Pacific Islands, and was recently detected in the eastern United States (Zhao et al., 2021), and Turkey (Keskin and Doi, 2025). The introduction and establishment of this tick, into North America was first confirmed in 2017 (Rainey et al., 2018). As of March 2024, *H. longicornis* populations are established in nineteen states in the eastern United States (APHIS-USDA, 2024). Examination of archival field collected specimens revealed that *H. longicorni*s was already present in North America in 2010 (Beard et al., 2018). Genetic analyses established that at least three unrelated *H. longicornis* females were introduced into North America (Egizi et al., 2020). Habitat suitability modeling predicts the potential for geographic range expansion of a large part of North America (Namgyal et al., 2020) with major concerns focused on its capacity to transmit SFTS virus, spotted fever group rickettsiae (*Rickettsia japonica*) and *A. phagocytophilum* (Zhao et al., 2021). Although SFTS virus is not known to be present in North America, *H. longicornis* is a competent vector of Heartland virus, a close relative of SFTS virus, in the United States. Both the closely genetically related Heartland virus and SFTSV were discovered in 2009 (Brault et al., 2018). Significantly, *H. longicornis* in North America undergoes parthenogenetic reproduction producing large numbers of progeny (Beard et al., 2018). All parasitic stages bite humans (Guglielmone and Robbins, 2018). In addition, Turkey and surrounding countries, including parts of Europe and the Middle East, are likely to become the next countries to be invaded by *H. longicornis*. The other members of this genus are mostly exophilic three-host ticks. In Eurasia, *H. concinna* is endemic in wide areas from western Europe to far-east Asia including Japan. Many *Haemaphysalis* are present in mixed and deciduous forests in moist habitats. The adults often prefer to infest large mammals and domestic animals while the immature stages are often found on birds, small/medium sized mammals. *Haemaphysalis* species are also regarded as vectors of TBE virus, SFTS virus, and *R. japonica* (Rubel et al., 2018).

**The genus *Hyalomma*** is a medically important species present in Eurasia and Africa with 27 species (Guglielmone and Robbins, 2018). They develop in open ecosystems with relatively hot and dry climates, and at low altitudes (Keirans and Durden, 2014). They are two or three-host ticks, with larvae and nymphs often being endophilic and feeding on small vertebrates while adults occur on large ungulates, including livestock. Adults can bite humans, and the most common human biting *Hyalomma* species are *H. anatolicum*, *H. marginatum*, *H. aegyptium*, and *H. lusitanicum.* They often exhibit hunting behavior, and will actively pursue potential hosts (Bernard et al., 2022). Due to climate change, this tick has expanded into south-western Europe, likely introduced into new geographic areas by migratory birds, and is considered an invasive species there. It is now established in the south of France and in Spain and it is responsible for human cases of Crimean Congo Hemorrhagic Fever (CCHF) (Bernard et al., 2024; Portillo et al., 2021). In a model of CCHF virus expansion in the Western Palearctic, Estrada-Peña et al. (2013) established that risk is associated with variations in temperature and host presence. These two factors have a direct impact on the development and survival of infected ticks. In Spain, the first human CCHF cases appeared in 2016, since then 17 cases have been reported with four cases in 2024. In Portugal, the first death associated to CCHF occurred during July 2024 (Zé-Zé et al., 2025).

## Main hosts/reservoirs of ticks

Ticks are strictly hematophagous ectoparasites which rely on various vertebrate hosts for their bloodmeal including mammals, birds, and reptiles. Ticks rely on their hosts for dispersal due to their small size and inability to fly. This makes the host-tick relationship crucial for tick survival and spread. Some of these tick host vertebrates are reservoirs of pathogens and maintain enzootic cycles of tick-borne pathogens. Humans are generally considered as incidental hosts (Eisen and Eisen, 2018; Nepveu-Traversy et al., 2024; Rizzoli et al., 2014). Ticks exhibit varying degrees of dependence on individual hosts and can be separated into one-host ticks (those which spend their entire life on a single individual host without leaving it to shed their cuticle between instars) (e.g., *R. microplus*), two-host ticks (e.g., *Hyalomma scupense*) which often remain on an individual host through both the larval and nymphal stage, only leaving once the nymph has engorged, and three-host ticks (e.g., *I. ricinus*), which leave the host between each instar (Leal et al., 2020). Additionally, ticks exhibit varying degrees of specificity for their hosts. Tick species can be associated with a single host species (monoxenic) (e.g., *Haemaphysalis pentalagi* and its rabbit host *Pentalagu funessi*) (Kwak et al., 2024b), a small number of host species (oligoxenic) (e.g., *Amblyomma nitidum* and *Laticauda* spp. snakes) (Kwak et al., 2020), or a wide range of host species (polyxenic) (*I. ricinus*) (Humair and Gern, 2000). Throughout different stages of development, some ticks exhibit distinctive changes in host specificity. It is well-known that ticks have established moderate to strong associations with specific wildlife as their primary hosts.

**Small mammals, especially rodents** (order Rodentia), are known as major reservoirs of zoonotic pathogens due to their high diversity. Those involved in tick-borne disease maintenance live in close association with humans (Keesing and Ostfeld, 2024). They are important hosts for larval and nymphal ticks (Eisen, 2022; Földvári et al., 2016; Furuno et al., 2017). One of the best example is Lyme borreliosis where rodents play a role as a host for *Ixodes* larvae and as reservoir for *B. burgdorferi* s.l. (Keesing and Ostfeld, 2024). In the northeastern United States, the most important rodent is the white-footed mouse (*Peromyscus leucopus*); in Europe, small rodents such as *Apodemus*, *Myodes*, *Microtus* (Gern and Humair, 2002; Rizzoli et al., 2014) and in Asia *Apodemus* is also a genus well-represented (Hou et al., 2014; Margos et al., 2013; Taylor et al., 2013). These rodents are also reservoirs of anaplasmosis, neoerhichiosis, TBE, relapsing fever associated to *Borrelia miyamotoi*, and babesiosis to name a few (Eisen, 2023; Rizzoli et al., 2014; Zhao et al., 2021). The complex nature of multiple pathogen reservoirs, tick vectors, and enzootic cycles is evident in North America where groundhogs (*Marmota monax*) are the main reservoirs for Powassan virus followed by *Peromyscus leucopus* with *Ixodes cookei* and *I. scapularis* as the primary vector ticks (Lange et al., 2023). Furthermore, tick density on rodents may differ from year to year as well as by the abundance of other hosts. Nymphal tick density on rodents increased by the exclusion of deer (Perkins et al., 2006).

Circulation of tick-borne pathogens is linked to other small mammals, such as **insectivores, erinaceomorpha, and lagomorpha** that can serve as hosts for all tick stages and a wide variety of tick species. In Europe, *Erinaceus* (hedgehog) is a good host for *Ixodes* and *Dermacentor* and reservoir for Lyme borreliosis, anaplasmosis, and TBE virus (Rizzoli et al., 2014). Its role as reservoir of pathogens in urban and peri-urban areas has been clearly shown in Europe for *N. mikurensis*, *A. phagocytophilum*, and *B. burgdorferi* s.l. (Földvári et al., 2014; Rizzoli et al., 2014). Lagomorphs are also an important tick host as the reservoir of *Francisella tularensis* and CCHFV (Bernard et al., 2022; Sharma et al., 2023). In East Asia, *I. ovatus*, *Ha. longicornis*, *Ha. flava, R. haemaphysaloides*, and *I. sinensis* are common ticks collected from hares (*Lepus* spp.) (Zheng et al., 2011).

**Large-sized mammals** especially wild ungulates such as deer and boars represent about half of the combined biomass of terrestrial wild mammals (Greenspoon et al., 2023). Historically, ruminant populations have decreased with human expansion (Chen et al., 2019; Fish, 2022). However, the populations of some deer (Côté et al., 2004; Fish, 2022; Iijima et al., 2023) and wild boar (Massei et al., 2015) are recently increasing. Because of their large biomass and population increase, the increase of large mammals has been suggested to be related to tick increase. The higher abundance of white-tailed deer (*Odocoileus virginianus)* (Kilpatrick et al., 2014; Ostfeld et al., 2018), sika deer (*Cervus Nippon*) (Iijima et al., 2022), roe deer (*Capreolus capreolus*) (Jaenson et al., 2012b), and wild boar (*Sus scrofa)*, (Doi et al., 2021; Ferroglio et al., 2024) are responsible for the greater abundance of tick species in the geographic regions where these large mammals occur. It is suggested that deer presence was more important on tick abundance than deer density. Indeed, in a study performed in dutch forests, the density of ticks did not increase with abundance of deer but experimental exclosure of deer significantly reduced tick population on a period of 2 years (Hofmeester et al., 2017). Wild boars contribute less to the maintenance of tick populations, especially for *Ixodes* ticks. In Europe, they harbor less ticks than cervids (Fabri et al., 2021; Hrazdilová et al., 2021). In contrast, *Dermacentor* on wild boar contribute to the circulation of *Rickettsia* in Europe (Sgroi et al., 2021). The expansion of wild boar in Japan resulted in an increase in the number of patients with tick-bites caused by *A. testudinarium* (Shimada et al., 2024). In United States, deer are critical as a blood meal source for adult *I. scapularis* to maintain the life cycle. They are also reservoir for *Ehrlichia chaffeensis* and *E. ewingii* (Eisen et al., 2017).

**Birds** play a role in the long distance dissemination of ticks and associated pathogens (Buczek et al., 2020; Estrada-Peña et al., 2021; Pitó et al., 2024). Ticks especially prefer to infest ground feeding birds such as *Erithacus rubecula*, *Turdus merula, Fringilla coelebs*, and *Passer domesticus* (Bacak et al., 2024; Wilhelmsson et al., 2020). Migratory birds coming from Africa with *Hyalomma lusitanicum* allowed the introduction of CCHV in new regions South of Europe, including Spain, Portugal and France (Bernard et al., 2022; Portillo et al., 2021; Zé-Zé et al., 2025). *Ixodes uriae*, *I. pavlovskyi*, *I. philipi*, *I. lividus*, *I. turdus, H. concinna* have a wide distribution within Eurasian, in part due to their association with birds. In Asia, the immature stage of *I. ovatus*, *I. persulcatus*, and *Ha. wellingtoni* are common examples of bird infestation ticks (Byun et al., 2024; Kwak et al., 2024a).

Migratory bird flyways that cross Asia, Europe, and North America converge in the arctic which is warming at a rate four times faster than the rest of the world (Rantanen et al., 2022). What impact might this warming trend have on ticks that are transported into the arctic on migratory birds in regard to survival, interactions with arctic fauna, and advancement to the next life cycle stage? Novel exchanges of tick species with their associated pathogens might occur that allows them to be introduced into different migratory bird populations and different migratory pathways, resulting in their introductions into new geographic areas. This is an area that should be investigated and further supports the importance of establishing tick and associated pathogen surveillance networks (Buczek et al., 2020; Pitó et al., 2024).

Multiple *Amblyomma* species are associated with **reptiles**. Common reptile ticks in Asia include *A. geoemydae, A. nitidum, A. helvolum*, and *A. varanense* which are also ticks reported with human infestations. In Turkey, *Hy. aegyptium* is the well-known tortoise tick which harbor CCHF virus. The tortoise could participate in the cryptic circulation of the virus with a potential transmission of CCHFV to humans (Kar et al., 2020). In Europe, some lizards including *Lacerta agilis* are host for *I. ricinus* nymphs and are reservoir for *Borrelia lusitaniae* but not for other Lyme associated *Borrelia* species (Tijsse-Klasen et al., 2010).

Invasive non-native hosts can also become novel hosts for local ticks as well as introduce “foreign” ticks into a new region. Doi et al. (2024) showed that introduced raccoons (*Procyon lotor*) and masked palm civets (*Paguma larvata*) are infested by Japanese tick species. Klink et al. (2024) also reported European Ixodid ticks were found on introduced raccoons and raccoon dogs (*Nyctereutes procyonoides*) in Germany (Byun et al., 2024). The eastern chipmunk (*Tamias striatus*) a rodent particularly present in United States has been introduced into France (Marchant et al., 2017) and Italy (Mori et al., 2018). It is an excellent host for ticks and reservoir of Lyme borreliosis (Keesing and Ostfeld, 2024), as such, it could modify the dynamic of transmission of tick-borne pathogens in Europe.

In recent years, the importance of host biodiversity in vector-borne diseases expansion has been suggested (Keesing et al., 2006; Keesing and Ostfeld, 2021). The complexity of the enzootic cycle of Lyme disease with different reservoir hosts, the major role of the white-footed mouse (*Peromyscus leucopus*), the deer population, and the concept of vector competence for optimal *Borrelia* circulation was suggested (Matuschka and Spielman, 1986). A diverse assemblage of vertebrates reduces the risk of Lyme disease in human (Ostfeld and Keesing, 2000). Based on these results, the concept of the dilution effect, i.e., that the presence of vertebrate hosts with a low capacity to infect food vectors (incompetent reservoirs) dilutes the effect of highly competent reservoirs, thus reducing the risk of disease, was suggested (Schmidt and Ostfeld, 2001; LoGiudice et al., 2003). The authors stressed the importance of biodiversity conservation in the face of anthropogenic activities affecting the host community in this type of zoonosis. However, this model has subsequently been questioned, and the effect of different host communities may in fact be a dilution or amplification of tick populations depending on competition between hosts, contact between hosts and ticks and host resistance to tick bites (Ogden and Tsao, 2009; Randolph and Dobson, 2012). The various models proposed show that the host community is not sufficient to predict risk, and that additional parameters, including temperature and vegetation, need to be included in the models (Kilpatrick et al., 2017).

## Tick-borne diseases on each continent

Due to tick population expansions, tick-borne diseases (TBDs) are also more frequently being diagnosed in humans in areas where those infections were seldom, if ever previously encountered (Table 1). Better awareness by clinicians and patients and improvements in diagnostic tools also contribute to the increase in TBD detections. However, TBDs continue to be underdiagnosed worldwide. They sometimes are introduced into new geographic areas which can complicate our understanding of the epidemiology of these diseases. Ticks transmit viruses, bacteria, and parasites to vertebrate hosts, and although they can harbor different potentially pathogenic microorganisms, co-infections are less frequently observed in patients than might be anticipated (Boyer et al., 2022). TBDs are zoonoses and humans constitute incidental, typically dead-end, hosts.

**FIGURE 1:**
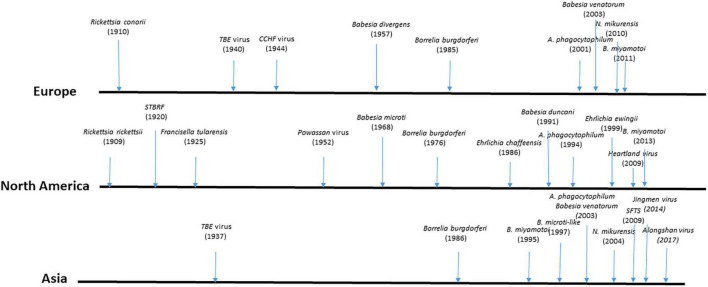
Main dates in the discovery of most important human tick-borne diseases. TBE, tick-borne encephalitis; CCHF, Crimean Congo Hemorrhagic Fever; STBRF, soft tick-borne relapsing fever; SFTS, Severe Fever Thrombocytopenia Syndrome.

Tick-borne diseases have been known since the second half of the 19th century; new ones are regularly identified and their medical importance is constantly growing (Figure 1). They are increasing in diversity and occurring in greater numbers due to climate change and socio-ecosystemic modifications (Madison-Antenucci et al., 2020; Pfäffle et al., 2013; Wikel, 2022). In North America, presently the United States is experiencing the greater burden of TBDs, and due to climate change Canada is becoming increasingly impacted by these diseases (Wikel, 2022). In Europe, expansion of TBDs to the North is observed especially for Lyme borreliosis, and TBE due to climate change (Lindgren et al., 2000). In south-western European countries such as Spain and France, the introduction of *Hyalomma* ticks, probably via migratory birds from Africa, has led to the appearance of human cases of CCHF in Spain (Portillo et al., 2021) and the detection of the virus in ticks in France (Bernard et al., 2024). At present, Asia seems to be more impacted by the emergence of new zoonotic tick-borne viruses, such as SFTS virus (Zhao et al., 2021). Ticks and tick-borne pathogens are increasingly transboundary threats. Lyme borreliosis is the most common vector-borne disease in the northern hemisphere and it has steadily increased in incidence and geographical range in many regions of Europe and North America (Eisen and Eisen, 2018; Eisen et al., 2016; Goren et al., 2023; Kugeler et al., 2021).

We review the current status of the main TBDs of the northern hemisphere, bearing in mind that they do not all present the same risk in terms of exposure, transmission, and infection. Transmission is linked to the duration of attachment of the infected tick, which differs according to the pathogen: *B. burgdorferi* sensu lato optimal transmission at 24 h with many more at 48–72 h, *Babesia microti* (36 h or more), *Francisella* (48 h), *Ehrlichia* and *A. phagocytophilum* (starting at 24 h), almost immediately for viral infections (de La Fuente et al., 2007; Eisen, 2018; Richards et al., 2017). Treatments are available for tick-borne bacterial and parasitic infections that are currently free of antibiotic resistance, the situation is more critical for tick-borne viral infections (Nepveu-Traversy et al., 2024), while vaccines against TBD are significantly lacking. Therefore, raising awareness among healthcare professionals and the general public about changing tick-borne disease threats is essential. Currently, the only vaccine available for TBDs in humans targets the TBE virus and two vaccines were approved in 2000 in Europe (Jaenson et al., 2018; Süss, 2003). More recently, a TBE virus vaccine was approved in the United States in 2021 (Hills et al., 2023).

The prevalence of infection in ticks varies according to the pathogens, the local environment, the tick stage, reservoirs, and the enzootic cycles: it can be as high in the case of some bacteria with up to 60% for *B. burgdorferi* s.s. in the United States (Maggi et al., 2019) while in Europe, an average prevalence of 16% in ticks has been recorded (Strnad et al., 2017). Tick infection is generally relatively low for viruses, often less than 1% (Randolph et al., 2000) and for parasites (Kumar et al., 2021).

In recent years, with the development of metagenomics, the characterization of vector microbiota has complicated our understanding of vector-pathogen-host interactions (Wang et al., 2023). New micro-organisms have been identified, some of which are simple endosymbionts such as *Coxiella*-type endosymbionts, *Rickettsia*-type endosymbionts and *Francisella*-type endosymbionts (Bonnet et al., 2017; Duron et al., 2018), others are potential pathogenic microorganisms like spiroplasma (Eimer et al., 2022; Matet et al., 2020). Symbionts are essential to tick survival as nutritional factors, but their role in the development of pathogens within the tick and in the transmission of pathogenic microorganisms to the vertebrate host is increasingly being described (Boulanger et al., 2023; Narasimhan et al., 2021).

Another important point to keep in mind in vector-borne diseases is that the detection within a tick of the molecular signature of a pathogen does not establish vector competence of that tick species for that pathogen (Eisen, 2020b; Kahl et al., 2002).

### Bacterial infections

Spirochetes include the genera *Treponema*, *Leptospira* and *Borrelia.* Within the genus *Borrelia*, two types of spirochete caused tick-borne diseases can be diagnosed in humans: Lyme borreliosis and relapsing fevers.

**Lyme borreliosis (LB)** is the most well-known, and an extensively well-studied TBD. It is the most commonly encountered vector-borne disease of the temperate zones of the Northern hemisphere. This bacterial infection is caused by six spirochete genospecies among the 20 validated genospecies (Table 1). The bacteria are maintained in a complex network of *Ixodes* vectors and different reservoir hosts, mainly rodents and birds (Wolcott et al., 2021). The vectors belong to the *I. ricinus* complex and the pathogens are extracellular spirochetes. Twenty-one species of *B. burgdorferi* sensu lato have been identified with three major pathogenic species: *B. afzelii* and *B. garinii* in Eurasia and *B. burgdorferi* sensu stricto mainly in North America (Stanek et al., 2012). First identified in Connecticut on the East coast of the United States in 1975, its presence was already suspected in Europe at the end of the 19th century (Lenormand et al., 2016).

In Europe, the incidence rate is ∼22 cases per 100,000 people, the actual number is most certainly higher, because LB is not notifiable in all European countries (Steinbrink et al., 2022). In the USA, it is a notifiable disease and based upon laboratory data is responsible for an estimated 476,000 human cases annually (Borchers et al., 2015; Nepveu-Traversy et al., 2024). In Asia, China reported its first case of LB in 1986. A meta-analysis of LB seroprevalence from 2005 to 2020 documented the presence of the disease in 29 provinces across China, with a core zone of endemicity in the northeastern provinces (Stark et al., 2022). In Japan, the disease was also identified for the first time in 1986. It is mainly reported from Hokkaido, the northernmost prefecture of Japan (Yamaji et al., 2018). Lyme borreliosis has been reported from other Asian countries including Korea, Taiwan, Nepal, Indonesia, and Malaysia. Human disease is characterized by a multisystemic disorder affecting the central nervous system with facial nerve palsy as a common clinical manifestation; the joints, heart, and/or skin can also be affected. The early manifestation may be a localized erythema migrans in around 80% of patients which occurs 3–30 days after the bite of an infected tick (Stanek et al., 2012). Some clinical manifestations such as lymphocytoma, late neuroborreliosis, and acrodermatitis chronica atrophicans are reported in Europe, due to different *Borrelia* species (Steinbrink et al., 2022). A vaccine candidate based on different OspA serotypes is currently in a phase 3 clinical trial (Bézay et al., 2024; Ghadge et al., 2024). It relies on a transmission-blocking process of the bacteria within the tick involving OspA (Wormser, 2022). OspA is a major antigen expressed by *Borrelia* in the *Ixodes* midgut (Pal et al., 2000).

**Relapsing fever (RF)** is caused by multiple *Borrelia* species and most commonly associated with transmission by Argasid, soft ticks, of the genus, *Ornithodoros* (Supplementary Table 1). These RFs are mainly present in Africa, the Middle-East and the Americas where different *Borrelia* species are associated to specific tick species (Jakab et al., 2022; Talagrand-Reboul et al., 2018). More recently, a new RF *Borrelia* transmitted by *Ixodes* was described: *B. miyamotoi*. First discovered in the hard tick *I. persulcatus* in Japan in 1995, it was thereafter identified in patients in Russia (2011), the United States (2013), and Europe (Madison-Antenucci et al., 2020). Its prevalence in hard ticks of the *I. ricinus* complex is not negligible but human cases are still limited. An extensive analysis determined the average incidence of *B. miyamotoi* infection of questing *I. ricinus* (1.0%), *I. scapularis* (1.1%), *Ixodes pacificus* (0.7%), and *I. persulcatus* (2.8%) with infection rate variations within a species (Burde et al., 2023). During 2022 and 2023, number of human *B. miyamotoi* infections reported to the Connecticut State Public Health Department were 7 and 4, respectively, which is likely fewer cases than actually occurred (Department of Public Health, 2025). Clinical symptoms are similar to *A. phagocytophilum* with fever and elevated liver enzymes (Madison-Antenucci et al., 2020). In immunocompromised individuals, the disease can be neuro-invasive (Sprong et al., 2018). Its reservoirs are mainly small rodents, *Peromyscus*, *Apodemus*, and *Myodes* species, and birds (Cleveland et al., 2023; Krause et al., 2015).

Bacteria of the Rickettsiales order are small obligate intracellular microorganisms transmitted by arthropod vectors (Londoño et al., 2023). As human pathogens, they possess two families, *Rickettsiaceae* responsible for rickettsiosis and *Anaplasmataceae* responsible for ehrlichiosis, anaplasmosis and neoehrlichiosis.

**Rickettsioses** have a worldwide distribution with a large variety of hosts and arthropods involved in their transmission. They can cause life-threatening diseases and are transmitted either by ticks (spotted fever group) or by non-tick vectors such as mites, fleas, and lice (Merhej et al., 2014). Rickettsiae are obligate intracellular bacteria infecting endothelial cells and macrophages and the pathogenesis of these infections was the subject of recent excellent reviews (Blanton, 2019; Helminiak et al., 2022; Sahni et al., 2019). In North America, *R. rickettsii* is transmitted by ticks (*Dermacentor, Amblyomma* and *Haemaphysalis)* and by *R. sanguineus* in Mexico and Brazil. *Rickettsia rickettsii* is responsible for RMSF, characterized by fever, headache, and rash. It can be deadly if not treated by appropriate antibiotic therapy. In Europe, *R. conorii* transmitted by *R. sanguineus* is responsible for Mediterranean spotted fever and *R. slovaca* and *R. raoultii*, responsible for TIBOLA (tick-borne lymph-adenopathy) is transmitted by *D. marginatus* and *D. reticulatus* (Oteo and Portillo, 2012). In China, *R. heilongjiangensis* and *R. raoultii* are the most widely distributed spotted fever group rickettsiae (Zhao et al., 2021) whereas in Japan, *R. japonica*, *R. heilongjiangensis*, and *R. tamurae* cause spotted fever (Imaoka et al., 2011); other human infectious rickettsial species are also present (*R. monacensis* and *R. helvetica*) (Thu et al., 2019). Rickettsial interactions with tick vectors and vertebrate hosts are being advanced by genome characterizations, functional genomics, and molecular studies (Kim, 2022).

**Human granulocytic anaplasmosis (HGA)** was first discovered in 1994 in the United States. It is caused by the Gram-negative bacterium, *A. phagocytophilum*, which displays an intracellular tropism for neutrophils. Vectors are members of the *I. ricinus* complex and the disease is present in Europe, North America, and Asia (Bakken and Dumler, 2015). This bacterial infection affects both domestic animals and humans. Infection can be asymptomatic or cause non-specific febrile illness with greater risk of morbidity and mortality in immunocompromised patients (Madison-Antenucci et al., 2020). A recent systematic literature review addressed clinical, diagnostic, therapeutic and epidemiologic aspects of infections with this increasingly important tick-borne pathogen (Schudel et al., 2024). Different *A. phagocytophilum* ecotypes have been identified that exhibit different host ranges. All human cases belong to ecotype I in Europe (Jahfari et al., 2014). There are two distinct variants of *A. phagocytophilum* in North America with the Ap-ha (human active) variant causing illness in humans and domestic animals and associated with a rodent reservoir (white footed mice, Eastern chipmunks) while the Ap-V1 variant is associated only with white tailed deer and considered to be non-pathogenic (Aardema, 2023; Keesing et al., 2014). North American *A. phagocytophilum* is genetically less diverse than those in Europe, suggesting an introduction of this organism from Europe (Aardema, 2023). The overall HGA prevalence worldwide is 8.13% with the highest incidence in humans at 11.07% occurring in North America, with occupationally exposed populations at greatest risk of infection (Karshima et al., 2023). There are several other *Anaplasma* species with zoonotic significance. In Cyprus, *Anaplasma ova* was detected in a human with fever after a tick bite (Chochlakis et al., 2010). In China, *Anaplasma capra* was detected in patients with febrile presentation associated with a history of tick bite (Li et al., 2015).

**Neoehrlichiosis** is caused by the Gram-negative bacterium*, N. mikurensis* (Portillo et al., 2018). The bacterium is intracellular, developing in the cytoplasm of endothelial cells. It was first discovered in brown rats, *R. norvegicus*, and the tick *I. ovatus* in Japan (Kawahara et al., 2004). The first clinical case was subsequently described in Sweden in an immunocompromised patient with thromboembolic complications (Welinder-Olsson et al., 2010). Presently, the disease is described as an infection with fever, rash, and thromboembolic complications in both immunocompromised and immunocompetent individuals (Portillo et al., 2018; Silaghi et al., 2016). In Europe, rodents (*Apodemus* and *Myodes*) are reservoirs and the level of *Ixodes* tick infection varies from 0.1% to 24.3% (Portillo et al., 2018). The disease has also been described in Asia (Zhao et al., 2021).

**Ehrlichioses** caused by obligate intracellular Gram-negative bacteria of the order Rickettsiales were considered veterinary pathogens until 1954 when human infection with *Rickettsia sennetsu* (later reclassified as *Ehrlichia sennetsu*, now *Neorickettsia sennetsu*) was identified in Japan (Gygax et al., 2024). Medical importance of *Ehrlichia* changed significantly with the identification of *E. chaffeensis*, causative agent of human monocytic ehrlichiosis (Paddock and Childs, 2003). Subsequently, *E. ewingii*, closely related to *Ehrlichia canis*, was found to infect humans (Adams et al., 2025; Gygax et al., 2024). Both *E. chaffeensis* and *E. ewingii* are transmitted by *A. americanum*, whose larvae, nymphs, and adults readily blood feed on humans and whited tailed deer, reservoir for these pathogens (Gygax et al., 2024; Paddock and Childs, 2003). *Ehrlichia muris* subsp. *eauclairensis* is a rarely encountered human pathogen whose primary reservoir is *Peromyscus leucopus* and vector is *I. scapularis* (Gygax et al., 2024; Xu et al., 2023). Human infections with other *Ehrlichia* species (*E. muris, E, canis*, and *E, ruminantum*) are rare (Gygax et al., 2024). Human monocytic ehrlichiosis almost entirely occurs within the United States; however, human ehrlichial infects have been rarely reported in Africa, Asia, and South America (Gygax et al., 2024). The clinical presentation, diagnostic, laboratory, and epidemiologic aspects of human monocytic ehrlichiosis are subjects of recent reviews (Gygax et al., 2024; Ismail and McBride, 2017) as is the molecular pathogenesis (Rikihisa, 2015).

**Tularemia** is a clinical entity caused by the Gram-negative, pleiomorphic, catalase-positive, non-motile coccobacillus *Francisella tularensis*, one of the most virulent bacteria known that is infectious at extremely low doses (Eickhoff and Blaylock, 2017; Sharma et al., 2023). Of the two important subspecies of this important pathogen, *Francisella tularensis* Type A (subspecies *tularensis*), the more virulent of the two, occurs exclusively in North America commonly associated with wild rabbits and hares. It can infect a diverse range of animal species (Keim et al., 2007). The second one, Type B (subspecies *holartica*) occurs in Europe and Russia is associated to rodents (Gray et al., 2024). *Francisella tularensis* is one of the most adaptable microbes to environmental conditions, hosts, and vectors with transmission cycles linked to differing ecologies of a Type A terrestrial cycle and Type B aquatic cycle (Carvalho et al., 2014). *Francisella tularensis* is resistant to environmental stresses and able to survive in soil, water, and carcasses for weeks (Carvalho et al., 2014).

Human infection occurs by direct contact with infected animal tissues or blood; indirectly by consumption of contaminated food, water, or inhalation of bacteria contaminated aerosols; and by the bites of infected ticks and tabanids (Carvalho et al., 2014; Sharma et al., 2023). Tularemia most frequently occurs during late spring, summer, and autumn with men more frequently infected than women (Ellis et al., 2002). Clinical presentation and course of tularemia depends upon virulence of bacterial strain, route of infection, infectious dose, host immune status (Nigrovic and Wingerter, 2008; Oyston, 2008). Incubation period post-infection is typically three to 5 days; however, it can range up to 20 days (Sharma et al., 2023). Clinical presentations include ulceroglandular, glandular, oculoglandular, orophyrangeal, and pneumonic with the ulceroglandular and glandular forms the most commonly occurring in North America with tick bite as the route of transmission (Eickhoff and Blaylock, 2017; Nigrovic and Wingerter, 2008; Sharma et al., 2023).

*Francisella tularensis* has been bioengineered and weaponized to be a significant agent of bioterrorism due to its high virulence, extremely low infectious dose and significant threat posed by aerosol delivery (Dennis et al., 2001; Oyston et al., 2004).

**Q fever or coxiellosis** is due to *Coxiella burnetii*, an obligate intracellular Gram-negative bacterium that infects a wide variety of mammals, birds, reptiles, and arthropods. It occurs over a wide geographic range with the exception of New Zealand and Antarctica as an environmentally stable microbe. It has a low infective dose of less than ten bacteria (Celina and Cerný, 2022). It is a zoonosis with the most common reservoirs being cattle, sheep, and goats (España et al., 2020). Infections in humans and farm animals occur predominantly due to inhalation of wind distributed spore-like (pseudospores) organisms (España et al., 2020). Davis et al. (1938) isolated a microorganism, later identified as *Rickettsia burnetii* and now known as *C. burnetii*, from the Rocky Mountain wood tick, *Dermacentor andersoni*, in western Montana. Although ticks are competent vectors of *C. burnetii*, they and other arthropods play minor roles in pathogen transmission to humans; however, ticks are likely more important in maintenance of the bacteria among other animal species in nature in a sylvatic cycle (Celina and Cerný, 2022; McQuiston et al., 2002). *Coxiella burnetii* is maintained in the infected tick population throughout the life cycle by both transovarial and transstadial transmission (Celina and Cerný, 2022). Additional arthropod hosts and sources of infection of vertebrate species are sucking lice and mites. Infection can also be acquired by consumption of unpasteurized milk and milk products as well as environmental exposure to infected placentas, aborted tissues, urine, and feces (Celina and Cerný, 2022; McQuiston et al., 2002).

Detection of serum antibodies in cattle, goats, and sheep, the main reservoirs, does not necessarily correlate with shedding of *C. burnetii* as indicated by fewer than 10 percent of sheep and 35 percent of bovines that were actively shedding *C. burnetii* being seropositive (Eldin et al., 2017). Multiple strategies have been employed to develop a *C. burnetii* vaccine, but no effective and fully safe vaccine has been developed (Sam et al., 2023).

The resistance and the possibility of airborne transmission have led to this bacterium being classified in list 3 in terms of biosafety. *C. burnetii* is a significant potential bioterrorism agent (Oyston and Davies, 2011).

### Viral infections

More than 35 virus species from six different families are transmitted by ticks (Wu et al., 2024). These viruses are RNA viruses. Their incidence is increasing: some are expanding their geographic range such as CCHF, TBE, Powassan, Heartland, Bourbon, and SFTS viruses and new ones are regularly identified in China (Alongshan, Wetland, Xu-Cheng virus) and in Europe (Tacheng virus 1&2) (Ergunay et al., 2023; Madison-Antenucci et al., 2020; Shah et al., 2023; Zhang et al., 2025). Because ticks can also transmit viruses transovarially to their offspring, ticks also are reservoirs of viruses in ecosystems (Moming et al., 2024; Nuttall and Labuda, 2003; Shah et al., 2023).

**Crimean-Congo hemorrhagic fever (CCHF)** is caused by infection with CCHF virus, an enveloped single-stranded negative-sense RNA virus belonging to the *Orthonairovirus* genus in the *Nairoviridae* family of the *Bunyavirales* order (Hawman and Feldmann, 2023). Seven genotypes with different virulence and different geographic range have been characterized (Bernard et al., 2024; Shahhosseini et al., 2021). Because of the risk of human-human transmission and the severity of the disease, CCHF virus is placed in biohazard class IV. It is the most medically important and widespread tick-borne viral disease, characterized by sporadic cases and outbreaks of hemorrhagic fever that occur over a broad geographic range from western China across Asia to southern Europe, the Middle East, and a vast area of Africa (Bente et al., 2013; Ergönül, 2006; Fereidouni et al., 2023). The geographic range of CCHF virus extends into southern Europe, likely due to the movement of migratory birds coming from Africa (Bernard et al., 2022; Portillo et al., 2021). It is mainly transmitted to humans by *Hyalomma* tick bites, but also through contact with blood or tissue of viremic animals during slaughtering, including human-to-human nosocomial transmission (Hawman and Feldmann, 2023; Shahhosseini et al., 2021). Most infections are subclinical. After a short incubation, it initially presents as prehemorrhagic and hemorrhagic stage with severe bleeding and ecchymosis and occasionally multiorgan failure. The case fatality rate of 5 to 30%. Differential diagnosis from other hemorrhagic fevers is essential (Madison-Antenucci et al., 2020)

**Tick-borne encephalitis (TBE)** is caused by a*n Orthoflavivirus* of the *Flaviviridae* family. This is a single-stranded RNA virus with a positive sense genome. Discovered in 1937 in the Far east of Russia, the virus is present only in Eurasia where three main virus clades occur: Far Eastern, Siberian, and Western European viruses. It is responsible for encephalitis with varying morbidity and mortality according to the virus type, the Far Eastern virus is the most lethal (30% case fatality) (Pustijanac et al., 2023). The main vector is *I. ricinus* in Western Europe and *I. persulcatus* in Asia; *D. reticulatus* and *Haemaphysalis* spp. are considered as secondary vectors of TBE virus (Chitimia-Dobler et al., 2019; Michelitsch et al., 2019). The infection rate of *I. ricinus* ranges from 0.1% to 5% and up to 40% for *I. persulcatus* in endemic areas of Siberia. Virus can also be transmitted by the consumption of unpasteurized milk or milk products derived primarily from goats exposed to infected tick bites (Pustijanac et al., 2023). Effective vaccines exist against TBE virus (Hills et al., 2023; Kunz, 2003).

**Powassan virus** is very closely related to TBE virus. It is classified into two genetically and ecologically distinct lineages: lineage I (POWV) mainly transmitted by *I. cookei* and lineage II (deer tick virus) mainly transmitted by *I. scapularis* (Shah et al., 2023). It spreads to people by the bite of an infected *Ixodes* tick. Although still rare, the number of reported cases of people ill from Powassan virus has increased in recent years. Most cases in the United States occur in the northeast and Great Lakes regions from late spring through mid-fall when ticks are most active. Increases in virus detection were recorded in Canada and Far East Asia (Madison-Antenucci et al., 2020). Initial symptoms can include fever, headache, vomiting, and weakness. Powassan virus can cause severe disease, including inflammation of the brain (encephalitis) or the membranes around the brain and spinal cord (meningitis). Symptoms of severe disease include confusion, loss of coordination, difficulty speaking, and seizures. The mortality rate is 15% (Shah et al., 2023).

**Severe fever with thrombocytopenia syndrome (SFTS)** is caused by a phlebovirus, named Dabie bandavirus, in the order Bunyavirales (Cui et al., 2024), isolated in central China in 2009 (Yu et al., 2011), and identified in Korea in 2012, Japan in 2013, and Vietnam in 2017 (Cui et al., 2024; Takahashi et al., 2014). The genome of the virus is a negative strand RNA. Spread of SFTS virus in East Asia, resulting in increased incidence of infections and the widespread distribution of the tick vector, indicates the expanding range of this tick-borne virus and its potential to become an increasing public health threat (Casel et al., 2021; Li et al., 2021; Liu et al., 2014; Seo et al., 2021; Yang et al., 2022). *Haemaphysalis longicornis* is the main vector of SFTS virus, with an infection rate of 8% (Casel et al., 2021; Cui et al., 2024). Clinically, SFTS is associated with fever, thrombocytopenia, leukocytopenia, multiorgan dysfunction, and a case fatality of 12-50% (Zhao et al., 2021). Human to human (Chen et al., 2013) and domestic cats (*Felis silvestris catus*) to human (Tsuru et al., 2021) direct transmissions have been reported. To date, SFTS virus has not been detected in *H. longicornis* populations introduced into North America. Animals with the highest positivity for the virus (infection rate and antibody detection) were mink (*Mustela siberica)* (91.11%) and pheasant (*Phasianus colchicus)* (42.86%). Better notification rate of SFTS, land use (Yasuo and Nishiura, 2019), favorable climate conditions (Iijima et al., 2025), and expansion of transmission with a potential role of migratory birds (Cui et al., 2024) might explain its higher rates of incidence in recent years.

**Heartland virus** disease was discovered in 2009 in the USA (Missouri) (McMullan et al., 2012). It belongs to the genus *Bandavirus*, family *Phenuiviridae*, order *Bunyavirales*. It is related to SFTS virus (Dembek et al., 2024). Pathogenesis of Heartland virus and SFTS virus infections share many features. Based upon serosurveys, Heartland virus occurs over a broad area of the eastern and midwestern United States. Heartland virus infection is likely under reported and expanding in geographic range (Dembek et al., 2024). *Amblyomma americanum* is a competent vector (Brault et al., 2018) and white-tailed deer a virus reservoir (Clarke et al., 2018). Significantly, *H. longicornis* is a competent vector for Heartland virus as well as transmit the virus transovarially to offspring (Raney et al., 2022). The current range of *H. longicornis* in North America is within a geographic area where Heartland virus transmission is established (Mantlo and Haley, 2023; Namgyal et al., 2020). Habitat range modeling for this species predicts a broad range in eastern North America from southern Canada to the coast of the Gulf of Mexico and an extensive region of the southern and midwestern United States that overlaps with Heartland virus range (Rochlin, 2019). This situation appears to be the confluence of all the factors needed to establish a robust enzootic cycle and spillover of human infections with a readily adapting and establishing invasive tick vector. Tick-borne disease implications of the invasion of *H. longicornis* into North America are unexpected and reveal the complexity of existing tick-host-pathogen ecologies and how those associations can be changed by introduction of an invasive tick species.

**Bourbon virus** is an emerging Thogotovirus also transmitted by *A. americanum* (Godsey et al., 2021; Lambert et al., 2015; Savage et al., 2017). Here as well, there are increasing concerns about the potential role of *H. longicornis* as a vector of Bourbon virus in North America due to discovery of Bourbon virus in field collected *Hae. longicornis* in Virginia (Cumbie et al., 2022).

Other viruses have been described as potential pathogens for humans but their occurrence is still rare and their impact on public health still limited. They belong to the families of *Flaviviridae* (Asian Omsk hemmorrhagic fever virus, Kyasanur Forest disease virus, Alkhurma virus, Louping Ill virus, Alongshan virus, Jingmen virus), Nairoviridae (Beiji Nairovirus, Tacheng Tick virus-1, Yezo virus, Songling virus, and Wetland virus), *Phenuiviridae* (Bhanja virus), *Orthomixoviridae* (Thogoto virus, Dhori virus, Oz virus and Bourbon virus), and *Reoviridae* (New World Colorado tick fever, Old World Eyach virus) (Ergunay et al., 2023; Moming et al., 2024; Wu et al., 2024). A novel member of the orthonairovirus genus of the Nairoviridae, Xue-Cheng virus, was identified from patients in northeastern China and detected in *H. concinna* and *Haemaphysalis japonica* ticks from the region (Zhang et al., 2025). Alongshan virus has been identified in different European countries (Ren et al., 2025).

### Parasitic infections

*Babesia* parasites are intraerythrocytic Apicomplexa, closely related to malaria parasites that are of vast veterinary and increasingly human medical importance with over 100 known species (Schnittger et al., 2012). *Babesia microti* is the most prevalent species in North America with *Peromyscus leucopus* reservoir and transmission by *I. scapularis* (Vannier and Krause, 2012). In Europe, *Babesia divergens* is transmitted by *I. ricinus* (Vannier and Krause, 2012). More recently, additional species have been described as human pathogens: *B. venatorum* in Europe and Asia, *B. duncani* in the USA, and a *B. crassa-*like species in China (Kumar et al., 2021). Typical symptoms include chills, fever, and fatigue with particular risks to immunocompromised patients. In the USA, awareness of babesiosis has increased in recent decades due to several factors including awareness by health workers and the public, expansion in deer population and *I. scapularis* tick population (Kumar et al., 2021). The co-infection of *B. microti*-*B. burgdorferi* s.s. in rodent reservoir provides a survival advantage to the parasites (Tufts et al., 2023). In Europe, the parasitic disease associated with *B. divergens* is less endemic with around 60 described cases but with a high mortality of 42% (Hildebrandt et al., 2021). In Asia, China is particularly affected by this parasitic infection with 4 species identified in human cases: *B. microti*, *B. divergens*, *B. venatorum* and a *B. crassa*-like (Zhao et al., 2021). The parasite is also present in India, Japan, Korea, and Mongolia (Kumar et al., 2021).

## One health: an integrated concept for effective control of TBDs

Based on the recognition of the role of wildlife in the emergence of many infectious diseases (Daszak et al., 2000), the “One Health” approach has become important for managing these diseases (Zinsstag et al., 2011), including TBDs (Dantas-Torres et al., 2012). One Health is based on the idea of building a sustainable relationship between human, animals (wildlife, domestic, and companion), and the environment (ecosystems) based on an understanding of disease ecology and pathogen transmission dynamics (Destoumieux-Garzón et al., 2018; Mackenzie et al., 2013). One Health extends the conventional framework in which drugs and vaccines have been the primary tools used to deal with the health of humans and domestic animals, separately. By taking this broader perspective, One Health aims to address infectious disease issues in a more comprehensive manner, including through surveillance, diagnostic tool development, disease and vector control strategies, wildlife and ecosystem management, and maintaining an appropriate ecological distance from wildlife. At the same time, One Health has a strong focus on strengthening linkages among environmental sciences, ecology, biosciences, human medicine, and veterinary medicine (Leung et al., 2012). The One Health framework has not been fully utilized in many cases, including in the control of TBDs, due to a lack of understanding of the dynamics underlying pathogen ecology and the environmental factors driving spillover, which has led to insufficient organization of ideas, direction, and collaboration among disciplines (Cunningham et al., 2017). Relationships among tick species, tick-borne pathogens, humans, vertebrate reservoir host, and tick amplification hosts are changing, creating new balances in relationships of these factors that amplify the need for increased studies of changing vector and disease ecology, development of novel control interventions for disease threat reduction, and information networks to ensure timely delivery of information to public health officials, medical and veterinary clinicians, advocacy groups, and the public – a One Health strategy in action.

To overcome this situation, the involvement of ecology and environmental studies, which have always been concerned with humans, other animals, and ecosystems, will be key to promoting collaboration among various disciplines and stakeholders (Destoumieux-Garzón et al., 2018; Garcia-Vozmediano et al., 2020; Ogden et al., 2019). One of the challenges is the lack of basic knowledge on the characteristics of species involved in transmission cycles, which is necessary for zoonotic disease risk assessment and prediction. In such a situation, pathogeography which analyses the relationship between the occurrence of disease and all potential factors of disease transmission cycle like, wildlife distribution, climate, land use, geography, and human population (Murray et al., 2018) is useful. Although pathogeography cannot clarify the mechanism of pathogen transmission, it suggests the important component of One Health that should be surveyed intensively (Iijima et al., 2025).

Zoonotic pathogen transmission cycles involve wildlife, domestic, and/or companion animal species, and are shaped by biotic and abiotic ecological processes at various spatial scales in addition to the community structure and the home ranges of the host and vector species involved. If the characteristics of each animal species, for example, as a host and/or reservoir, were known in such cases, the framework of community ecology and landscape ecology that takes into account species interactions and spatial processes would improve the accuracy of assessing and predicting infectious disease risk. Such basic findings have been gradually obtained in recent years (Ostfeld et al., 2018; Tatemoto et al., 2022), and future progress in ecological approaches is expected. Furthermore, given the transmission cycles of zoonotic pathogens crosses the boundaries of the natural environment and human managed habitats, it is difficult to implement One Health approach for a single administrative sector, research field, or legal system to take action that requires collaboration across different organizations and disciplines (Johnson et al., 2022; Machtinger et al., 2024). This would be one of the biggest challenges in expanding the options for tick-borne zoonosis control.

In the following parts, we raise the important issues that should be studied to promote the prevention of TBDs based on the One Health approach (i.e., human-animal-ecosystem relationships).

### Land use

Because forests harbor more ticks than other habitats (Bourdin et al., 2023), and are sites at higher risk of encountering emerging infectious diseases (Allen et al., 2017), deforestation or forest fragmentation affects the spillover of TBDs (Diuk-Wasser et al., 2020; Kilpatrick et al., 2017). Modification of ecosystems by humans sometimes increases populations of zoonotic pathogen reservoirs, such as rodents (Keesing and Ostfeld, 2021; Mendoza et al., 2020). Forest edges that are created by tree cutting provide a suitable habitat for deer (Miyashita et al., 2008), and can result in their increased abundance (Takarabe and Iijima, 2020). Forest fragmentation promotes tick abundance (Boulanger et al., 2024; Ehrmann et al., 2017; Ferrell and Brinkerhoff, 2018) and infection risk of TBDs (Iijima et al., 2025). The effect of ecosystem modification on the spillover of TBDs, however, is not simple. Mathematical models predicted that host community composition, not a single host abundance, may play a crucial role in explaining the variation in prevalence of tick-borne infections in hosts (O’Neill et al., 2023). The effect of land use on the spread of TBDs needs to be examined from several angles, including a multidisciplinary approach involving epidemiology, entomology, community ecology, and animal behavioral ecology.

### Invasive non-native vertebrate hosts

The invasion of non-native species is considered a major cause of biodiversity and ecosystem service loss, but it is also sometimes associated with an increased risk of zoonotic diseases, including TBDs. There are two ways in which invasive alien mammals can be involved (Chalkowski et al., 2018; Strauss et al., 2012): the introduction of new pathogens and ticks from non-native hosts (i.e., spillover process) or through the amplification of pathogens and ticks originally present in the ecosystem by non-native mammals (i.e., spillback process). For example, On Niijima Island, Japan, the introduction of sika deer allowed the expansion of the non-native ticks, *Haemaphysalis* (Doi et al., 2020). Non-native hosts can also amplify the dynamic of pathogen transmission as shown in Japan with invasive raccoon (*Procyon lotor*) for SFTS (Doi et al., 2021; Tatemoto et al., 2022), in France with chipmunk (*Tamias sibiricus*) and Lyme borreliosis (Marsot et al., 2013) or in east of Europe with invasive raccoon (*Procyon lotor*) and *A. phagocytophilum* (Hildebrand et al., 2018). In United States, exotic game ranches have become a major business in Texas since the 1950s, resulting in what is now a $1.3 billion industry with over two million exotic animals representing 135 species, predominantly of African and Asian origin (Pitchstone Waters, 2020). The second largest antelope in the world, nilgai (*Boselaphus tragocamelus*), was introduced into South Texas in 1930 for meat production. Nilgai population in South Texas in 2020 was estimated at 30,000 with thousands of animals roaming freely outside of enclosed ranches and occupying diverse habitats (Pitchstone Waters, 2020). A significant concern is the threat of bovine babesiosis transmitted by *R. microplus* and *R. annulatus* and the dispersal of these ticks by increasing numbers of free ranging nilgai (Lohmeyer et al., 2018; Sliwa et al., 2023).

### Ecosystem restoration

Currently, ecosystem restoration is a key issue in the conservation of biodiversity as the Kunming-Montreal Global Biodiversity Framework of the Convention on Biological Diversity ensures at least 30% of ecosystems are conserved or at least under restoration by 2030. However, careless conservation or restoration may cause the increase of TBDs risk by increasing host abundance. For example, density of infected ticks increased with the increase of landscape connectivity (Heylen et al., 2019). Same patterns have been observed in Europe and the United States due to the increase in host mammals and ticks in urban green areas and the associated increase in pathogens (Gassner et al., 2016; Rizzoli et al., 2014; VanAcker et al., 2019). Therefore, ecosystem restoration should be undertaken while also considering the impact of TBDs, and in some cases, reducing the abundance of important reservoir hosts like deer in close proximity to human habitat to reduce the risks of spillover, especially around urban areas (Millins et al., 2018).

### Modifications of hunting practices and socio-economic changes

Socio-economic changes have been drastic the last century. Deforestation and deer hunting reduce tick populations (Fish, 2022; Godfrey and Randolph, 2011; Matuschka and Spielman, 1986). In the 1930’s for the United States and in the 1940’s for Europe, deer expansion and reforestation occurred (Fish, 2022; Matuschka and Spielman, 1986). In present times, the population of hunters is decreasing in many developed countries due to changing demographics, interests, and urbanization (Manfredo et al., 2020, 2017). Population control of deer and boar by game hunting sometimes fails (Massei et al., 2015; Simard et al., 2013). The decrease of hunting pressure on wildlife and other actions to protect and restore nature cause not only increased deer abundance, but also the expansion of wildlife in urban areas and promote tick favorable habitats (Honda et al., 2018; Sprong et al., 2018). Urban wildlife directly brings ticks into human dominated areas (e.g., parks) that increase tick-bite risk for pets and humans accompanied by TBDs infection risk. Multidisciplinary teams should be built, including biologists, veterinarians, local stakeholders, sociologists, and others, to deal with population management of tick reservoir hosts (Garcia-Vozmediano et al., 2022; Valente et al., 2020).

### Medical aspect

It is reasonable to assume that several medical related factors contributed to the high incidence of emerging tick-borne diseases in recent years. Better awareness of the epidemiology, clinical manifestations, and diagnostic tools of TBDs have improved the detection and control of these diseases (Fang et al., 2015; Madison-Antenucci et al., 2020; Sprong et al., 2018). First, advances in sequencing technologies have made it feasible to obtain the sequences of previously unrecognized tick-borne pathogens (Tijsse-Klasen et al., 2014). Of note, the application of metagenomic approaches to clinical specimens has made it possible to detect pathogens without prior knowledge (Piantadosi et al., 2021). Furthermore, it is conceivable that improved therapeutic options and longer lifespan have increased the opportunities for immunosuppressed patients to be exposed to tick bites, resulting in clinical disease (Gynthersen et al., 2023). Finally, increased knowledge and awareness of TBDs among medical personnel could lead to the discovery of new tick-borne pathogens in patients with a history of tick bite (Eisen, 2022; Fang et al., 2015; Sprong et al., 2018).

## Prevention of tick bites and transmission of tick-borne pathogens in the context of tick range expansion

As described in this review, significant geographic range expansions are occurring among human biting tick species across the Northern hemisphere. Tick range expansions into new regions have the potential to introduce previously unencountered pathogens into those areas (Paules et al., 2018). From a public health perspective, range expansion creates the challenges of tick and pathogen surveillance to inform physicians, veterinarians, public health workers, and the public about emerging disease threats. Introduction of a tick species into a new region raises questions regarding changes in tick ecologies; balance and interactions among established and newly arriving tick species; and, the need for revised and novel strategies to achieve tick and disease control prevention (Eisen, 2020a; Molaei et al., 2019; Sonenshine, 2018; Wikel, 2022).

Factors influencing geographic range and population size are dynamic and dependent upon the complex interplay among macro and micro climate variations, vegetation patterns, land use, land fragmentation, habitat modifications (agricultural, residential, recreational), host animal diversity (domestic, wildlife, exotic species), human behavior, commerce, economics, government policies, population growth, population movement, and evolutionary changes occurring in ticks and tick-borne pathogens (Alkishe et al., 2021; Baneth, 2014; Dantas-Torres, 2015; Gebreyes et al., 2014; Pfäffle et al., 2013; Sonenshine, 2018). Each of these continually evolving factors, to differing degrees, influence tick and pathogen ecology, enzootic cycles of tick transmitted infectious agents, disease incidence, epidemiology, and selection of appropriate control approaches (Ogden et al., 2021; Sonenshine, 2018; Wikel, 2018).

Changing biotic and abiotic factors influence the tick-host-pathogen triad (Dantas-Torres, 2015; Eisen and Stafford, 2021). Tick biology, ecology, geographic ranges, and population densities are influenced by variations in temperature, humidity, soil moisture, vegetation, leaf litter, shade, and availability of pathogen reservoir and tick population amplification vertebrate species (Alkishe et al., 2021; Sonenshine, 2018; Springer et al., 2020). Changing climate is resulting in a warmer environment, vegetation stress, and greater aridity (Overpeck and Udall, 2020). Increasing temperatures will continue to be a determinant of changing tick population sizes and geographic distributions (Alkishe et al., 2021; Dantas-Torres, 2015). Decreased rainfall and resultant drier environment could increase tick mortality (Ogden and Lindsay, 2016). However, the influence of rain events of increased intensity and flooding on tick ecology are essentially unknown. Warmer temperatures can increase the seasonal duration of tick and human activities that result in increased potential for exposure to ticks and pathogen transmission (Gilbert et al., 2014).

What are the interactions that can and do occur when a tick species expands its range into new territory? The fact that the newly introduced tick survives and eventually thrives indicates that climate and habit factors are favorable in consideration of the fact that the vast majority of the life cycle of nearly all ixodid tick species is spent free living from vertebrate hosts. An excellent review by Phillips and Sundaram examines factors impacting ticks introduced into an existing community of resident tick species and vertebrate hosts and the subsequent impacts on tick community ecology, infection dynamics, and human diseases. These are under studied areas that deserve increased attention (Phillips and Sundaram, 2025).

Ticks expanding into a new geographic area or invasive ticks may contribute to tick-borne disease by bringing infectious agents with them and introducing “new” infectious agents into the region or by becoming a competent vector of currently existing pathogens in the new habitat (Chalkowski et al., 2018; Phillips and Sundaram, 2025). As described previously, an example of the second scenario is the introduction of *Ha. longicornis* into North America and the subsequent demonstration of the competence of this species as a vector of the North American Heartland and Bourbon viruses.

Consequently, human biting ticks and the disease causing agents they transmit are increasing public health threats due to geographic range expansion, increasing size of tick populations, emergence of newly recognized pathogens, introduction of invasive tick species that are resulting in part from changing weather patterns, land use modifications, biodiversity loss, and human activities/behaviors; all of which result in significant challenges for tick control and disease prevention (Alkishe et al., 2021; Dantas-Torres, 2015; Eisen and Paddock, 2021; Köhler et al., 2023; Tsao et al., 2021; Wikel, 2022). As a result of these evolving interactions and the resulting public health threats there exist critical needs to implement existing and develop novel tools and strategies to prevent tick bites, control tick populations, and reduce transmission of tick-borne pathogens. Achieving these objectives poses significant challenges.

Control of mosquitoes and mosquito-borne diseases is commonly a publicly funded community activity while prevention of tick bites and TBD transmission is primarily the responsibility of individuals for themselves personally and for their property (Burtis et al., 2024). Prevention of tick bites and TBDs is achieved by use of personal protective measures that include application of skin repellents, wearing of protective clothing that is either untreated or treated, performing tick checks, showering to remove unattached ticks, and, when possible, placing removed outdoor clothing in a dryer at high heat to kill unattached ticks (Eisen, 2022). Protection measures may vary between Europe and the United States, particularly with regard to the use of pyrethroids in the environment or for impregnating clothing (Boulanger, 2022). In France, a recent report indicates the risk of these pesticides to human health, particularly for pregnant women (Pesticides et santé-Nouvelles données (2021)). So, permethrin-treated clothing is no more recommended by the French Ministry of Health. Environmental suppression of tick populations is another approach for achieving tick bite and pathogen control; this approach is particularly implemented in United States (Stafford et al., 2017). Methods used include application of acaricides, biopesticides, biological agents that target host seeking ticks, landscape management, rodent reservoir targeted bait boxes that can deliver an oral vaccine, antibiotic, or acaricide, and methods that exclude, eliminate, or deliver acaricides to deer (Burtis et al., 2024; Stafford et al., 2017).

Risk of tick exposure varies across populations by age groups, occupation, recreational activities, and human behaviors (Eisen, 2022; Stafford et al., 2017). The peridomestic environment is an increasingly common place for contact with ticks and for acquiring tick transmitted infections, particularly recognized for Lyme borreliosis (Hansford et al., 2022; Stafford et al., 2017). Personal protective measures are the most commonly used methods for prevention of tick bites, but there are significant variations among individuals in regard to consistency of use and how applied (Eisen, 2022). Area wide tick control measures can be increasingly challenging based upon considerations of effectiveness, cost, required scale of application, lack of organizational implementation structure, and the changing geographic ranges of ticks and the pathogens they transmit (Eisen and Stafford, 2021; Sprong et al., 2018). Effectiveness of these interventions individually and collectively remains a topic of controversy and requires ongoing study to improve outcomes (Keesing et al., 2022). In addition, novel tick control tools are needed that can facilitate advances in integrated tick management, highlighting the multiple challenges of support for basic research, industry investment and commitment for commercialization, navigating a complex regulatory environment, and an uncertain market (Eisen and Stafford, 2021; Stafford et al., 2017).

Fundamental studies of tick biology and ecology are needed for many species of human biting ticks across broad, expanding geographic ranges that result in newly recognized, unstudied, interactions and relationship changes among multiple tick and host species and their associated human pathogens. Until such studies are conducted there will be a lack of data to inform public health officials, health care providers, and the public about tick bite and disease threats and the development of optimal strategies for their control.

Tick-borne diseases are zoonotic diseases with wildlife species being pathogen reservoirs and amplifying hosts that increase tick populations (Pfäffle et al., 2013). These zoonotic diseases involve complex enzootic cycles that include complex interactions at the interface among vertebrate hosts, tick vectors, climate, ecosystems, and humans (Gebreyes et al., 2014). Control and prevention of zoonoses require robust surveillance networks at the wildlife-human interface, vector biologists, physicians, veterinarians, public health experts, laboratorians, laboratory capacity, skilled personnel whose expertise encompasses disciplines of human and veterinary medicine clinical and basic research and related sciences – in essence a One Health approach (Dantas-Torres et al., 2012; Destoumieux-Garzón et al., 2018; Gebreyes et al., 2014). A One Health approach promoting synergies among multiple disciplines, agencies, and governments is central to advancing global health security in a rapidly changing world (Garcia-Vozmediano et al., 2022; Sinclair, 2019).
