# Supplementary material for: Ticks and tick-borne diseases in the northern hemisphere affecting humans
Source: Front Microbiol. 2025 Aug 8;16:1632832. doi: 10.3389/fmicb.2025.1632832 (PMC12370716; doi:10.3389/fmicb.2025.1632832)
Supplement: Supplementary file 1 [file Table_1.docx]

**Supplementary data - Table 1** : Main tick genera potentially biting human. After [(Eisen, 2022)](https://www.zotero.org/google-docs/?qIjHIp), (Negi, Kandari, et Arunachalam 2021)[(Guglielmone and Robbins, 2018)](https://www.zotero.org/google-docs/?3hlYDk)

| **Ticks** | **Asia: China/Japan** | **Europe** | **USA/Canada** |
| --- | --- | --- | --- |
| **Hard ticks** | | | |
| ***Amblyomma*** | *A. testudinarium*  *A. geoemidae*  *A. helvolum* | (-) | *A. americanum*  *A. maculatum* |
| ***Dermacentor* spp.** | *Dermacentor nuttalli*  *D. taiwanensis*  *D. auratus*  *D. silvarum*  *D. bellulus*  *D. marginatus* | *D. reticulatus*  *D. marginatus* | *D. andersoni*  *D. variabilis*  *D. occidentalis*  *D. albipictus* |
| ***I. ricinus* complex and *Ixodes* spp.** | *Ixodes acutitarsus*  *Ixodes persulcatus*  *Ixodes ovatus*  *Ixodes granulatus*  *Ixodes sinensis*  *Ixodes pavlovskyi*  *Ixodes tanuki*  *Ixodes monospinosus* | *Ixodes ricinus*  *Ixodes persulcatus*  *Ixodes hexagonus*  *Ixodes uriae* | *Ixodes scapularis*  *Ixodes pacificus*  *Ixodes cookei*  *Ixodes uriae* |
| ***Haemaphysalis* spp.** | *Haemaphysalis longicornis*  *Ha. flava*  *Ha. punctata*  *Ha. concinna*  *Ha. cornigera*  *Ha. japonica*  *Ha. hystricis*  *Ha. parva* | *Ha. punctata*  *Ha. concinna*  *Ha. hispanica*  *Ha. inermis*  Ha*.* parva | *Ha. longicornis* |
| ***Hyalomma* spp.** | *Hy. scupense*  *Hy. asiaticum* | *Hy. marginatum*  *Hy. lusitanicum*  *Hy. rufipes*  *Hy. aegyptium*  *H. scupense*  *Hy. anatolicum* | (-) |
| ***Rhipicephalus* spp.** | *R. sanguineus* sensu lato  *R. bursa* | *R. sanguineus* s.l.  *R. bursa*  *R. turanicus* s.l. | *R. sanguineus* s.l. |
| Soft ticks | | | |
| ***Argas* spp.** | *A. persicus*  *A. vespertilonis* | *Argas reflexus* | A*.monolakensis* |
| ***Ornithodoros* spp.** | *O. tartakovsky* | *O. capensis*  *O. maritimus*  *O. erraticus* | *Ornithodoros hermsi*  *Ornithodoros turicata*  *Ornithodoros parkeri* |
